# IFNλ1 is a STING-dependent mediator of DNA damage and induces immune activation in lung cancer

**DOI:** 10.3389/fimmu.2024.1525083

**Published:** 2025-02-12

**Authors:** Stine Høvring Godsk, Caroline Maren Stengaard Jensen, Trine Vilsbøll Larsen, Johanne Ahrenfeldt, Kristine Raaby Gammelgaard, Martin Roelsgaard Jakobsen

**Affiliations:** ^1^ Department of Biomedicine, Aarhus University, Aarhus, Denmark; ^2^ Department of Molecular Medicine, Aarhus University Hospital, Aarhus, Denmark; ^3^ Department of Clinical Medicine, Aarhus University, Aarhus, Denmark

**Keywords:** cancer immunology, STING, interferon lambda, NSCLC, CRISPR/Cas9

## Abstract

**Introduction:**

The importance of the cGAS-STING pathway and type I interferon (IFN) in anti-tumor immunity has been widely studied. However, there is limited knowledge about the role of type III IFNs in cancer settings. Type III IFNs, comprising IFNλ1-4, are opposite to type I IFN only expressed by a few cell types, including epithelial cells, and the receptor subunit IFNLR1, is equally only expressed on limited types of cells.

**Methods:**

Gene and protein expression of the cGAS-STING signaling pathway was characterized in a series of non-small cell lung cancer (NSCLC) cell lines. Herring-testis DNA stimulation and chemotherapy drugs (doxorubicin and cisplatin) were used to activate the cGAS-STING pathway, and the level of activation was determined by measuring changes in the transcriptomic profile as well as type I and III IFNs by ELISA. Re-expression of IFNLR1 on cancer cell lines was achieved using CRISPR activation (CRISPRa) followed by evaluating chemotherapy-induced apoptosis using flow cytometry assays.

**Results:**

STING was not broadly expressed across the NSCLC cell lines. Those cancer cell lines expressing all relevant factors supporting the cGAS-STING pathway secreted IFNλ following STING activation whereas only few of them expressed IFNβ. Treatment with chemotherapy drugs likewise preferentially induced IFNλ, which was abrogated in CRISPR-Cas9 STING knock-out cells. Expression of IFNLR1 was found downregulated in the cancer cell lines compared to the benign epithelial cell line Nuli-1. Rescuing IFNLR1 expression by CRISPRa in multiple cancer cell lines sensitization them to IFNλ-stimulation and resulted in significant reduction in cell viability.

**Conclusion:**

Downregulation of IFNLR1 can be an immune evasion mechanism developed by cancer cells to avoid responding to endogenous type III IFNs. Thus, rescuing IFNLR1 expression in NSCLC in conjunction to chemotherapy may potentially be harnessed to elevate the anti-tumoral responses.

## 1 Introduction

One of the hallmarks of activating the innate immune system is the production of interferons (IFNs) and inflammatory cytokines. The role of IFNs in cancer and how they support anti-tumor responses can be dated back to 1969, where it for the first time was shown to eradicate tumors in mice (1). Sixteen years later, type I IFN alpha was approved as the first type of immunotherapy to treat cancer though the mechanism of action was not clear. The diverse biological effects and high degree of toxicity did, however, rapidly limit the use of type I IFNs in clinical practice.

Today, we have a much more detailed understanding of how IFNs support antitumoral activities, and how this knowledge can be harnessed to elevate the effects of other more modern immunotherapies such as checkpoint inhibitors (2, 3). Importantly, the interest in IFNs role in modulating the immune system in cancer can partly be credited to the exploration of how innate immune pathways, and in particularly how the cGAS-STING DNA-sensing pathway (4, 5) is involved in anti-cancer biology (6, 7). Several clinical and preclinical studies have linked an active cGAS-STING pathway to superior positive outcomes for cancer treatment with chemotherapy, radiotherapy, and immunotherapy (6, 8–13). This most likely comes down to the mechanism of action being that accumulation of cytosolic DNA fragments in cancer cells are sensed by cGAS, which produces the small molecule 2’3’-cGAMP that binds STING and activate signaling, resulting in production of type I IFNs and selective inflammatory cytokines (14). The use of DNA-damaging therapy like radiotherapy and chemotherapy and their clinical effect can therefore be associated with STING expression and activation (15–18).

A few recent studies suggest that cancer cells can evade the cGAS-STING pathway simply by suppressing STING expression (19, 20) or by abrogating signals downstream the pathway (21). Impairment can also occur further upstream by hijacking genes involved in DNA repair and thereby concealing DNA damage and preventing micronuclei formation that otherwise would feed cytosolic DNA to the STING pathway (22–24). In contrast to this, STING pathway activation in cancer can be co-opted by tumors to promote their growth through the expression of immunomodulatory interferon stimulated genes (ISGs) like PD-L1 (25, 26), or through activation of non-canonical pathways activating NF-κB-dependent transcription of e.g. IL-6 that has been found to be associated with a pro-tumor response (27–29).

Downstream of STING pathway activation, CXCL10, CCL5 and especially type I IFNs are often used as surrogate markers for STING activity in tumors and cancer cells lines (16, 30). However, emerging evidence suggests that the much less studied type III IFNs are also produced via STING pathway activation and this group of cytokines might harbor great potential as a target in cancer therapy (31, 32). In humans, four isotypes of type III IFN (IFNλ1–4) exist. The IFNλs signal through a receptor complex composed of the IFNλ receptor 1 (IFNLR1) and the IL-10 receptor beta (IL-10Rβ) and utilize the JAK/STAT pathway to induce a broad array of ISGs (33, 34). A key feature of IFNλ signaling is the restriction of IFNLR1 expression to epithelial cells and certain immune cell subsets (34, 35). The native involvement of the IFNλ cascade in epithelial cells makes it a potentially important immunological pathway in NSCLC and other epithelial-derived cancers. IFNλ has been shown to reduce proliferation and induce cell apoptosis of cancer cells (36–39) and cancer cells overexpressing IFNλ show decreased tumor growth *in vivo* (40, 41). However, the induction of IFNλ-signaling downstream of STING activation, its regulation in NSCLC, and its role in eliciting a chemotherapeutic response have not yet been explored. Here, we report the regulation of STING-mediated IFNλ signaling in NSCLC, with a focus on IFNLR1 expression and the potential of chemotherapy induced IFNλ signaling as a therapeutic target in cancer treatment. We find that IFNλ serves as a better marker of STING pathway activation in epithelial cancer cells than IFNβ, and that cancer cell-specific downregulation of IFNLR1 prevents autocrine and paracrine IFNλ signaling. Additionally, we discover that CRISPR mediated transcriptional activation of IFNLR1 sensitizes NSCLC cell lines to IFNλ signaling leading to a reduced viability and supports chemotherapy induced apoptosis.

## 2 Materials and methods

### 2.1 Cell culture

All cell lines were purchased at ATCC (ATCC/LCG, Wesel, Germany) except PC9 (PHE culture collection, Salisbury, UK) and Nuli-1 (donated from Professor Christian Holm, Aarhus University, Denmark). Cells were grown in RPMI (Sigma-Aldrich, Cat#: R8758) or DMEM (Sigma-Aldrich, Cat#: D6429) according to supplied instructions supplemented with 10% fetal bovine serum (Sigma-Aldrich, Cat#: F9665), 1% L-Glutamine (Thermo Fisher Scientific, Cat#: 25030024) and 1% Penicillin-streptomycin (Thermo Fisher Scientific, Cat#: 15140122). Nuli-1 cells were grown in culture flasks precoated with collagen (Sigma-Aldrich, Cat#: C7521) in Bronchial Epithelial Cell Growth Basal Medium (Lonza, Cat#: CC-3171) with supplements and growth factors (Lonza, Cat#: CC-4175). The cells were grown at 37°C and 5% CO_2_.

### 2.2 Transfection

For all experiments including transfection, cells were seeded one day prior to transfection in a suitable plate size (Nunc 12-well, Nunc 24-well or Nunc 96-well plates). Lipofectamine® 2000 (Invitrogen, Cat#: 11668019) was used for transfection with 2μg/mL double stranded herring testis DNA (HT-DNA) (Sigma-Aldrich, Cat#: D6898-250) or 40ng/ml polyinosinic-polycytidylic acid (Poly I:C)-LMW (Invivogen, Cat#: tlrl-picw) according to the manufacturer’s instructions.

### 2.3 Chemotherapy treatment

For all experiments using cisplatin and doxorubicin, an approximated IC_50_ dose was used. Due to stability of the drugs, the exact concentration of the drugs corresponding to an IC_50_ dose varied throughout the course of the experiments. For cisplatin, the following dose ranges have been given to the individual cell lines; H358: 3-16μM, H1650: 3.34-8.5μM, H2228: 0.6-7.3μM, and H596: 1.6-8.5μM. For doxorubicin, the following dose ranges have been given to the individual cell lines; H358: 0.5-0.8μM, H1650: 0.5-1.1μM, H2228: 0.5-0.95μM, and H596: 0.5-1.1μM.

IC_50_ values were determined by seeding 5000-15000 cells in a Nunc 96-well plate and 24 hours later treating the cells for 48 hours with a dilution range of either cisplatin or doxorubicin. Viability as percentage of an untreated control was determined using CellTiter 96 AQueous One Solution Cell Proliferation Assay (Promega, Cat#: G3580) according to manufacturer’s instructions. A regression analysis was performed to estimate the IC_50_ value.

### 2.4 Reporter cell assay for type I IFN measurement

In **Supplementary Figures 1A, B**, type I IFN production was measured using a biofunctional Human HEK-Blue IFN-α/β reporter cell assay (Invivogen). Fifty microliters of standard (IFN-α standard curve starting at 1000U/mL (PBL Assay Science, Cat#: 11100-1)) or supernatants from the stimulated cells were added to the HEK-Blue IFN-α/β reporter cells overnight. Supernatant (20 μl) from HEK-Blue IFN-α/β reporter cells were then mixed with 180 μL QUANTI-blue (Invivogen, Cat#: rep-qb2) and the optical density at 620 nm was determined using a microplate reader to determine final concentration as U/mL.

### 2.5 ELISA

Detection of CCL5/RANTES, IFNβ, IFNλ, and IL-6 in supernatants was caried out using Human DuoSet ELISA (R&D systems, Cat#: DY278, DY814, DY7246, DY206) according to the manufacturer’s instructions. The optical density was determined using a microplate reader set to 450 nm and 570 nm. The readings at 570 nm were subtracted from the 450 nm-reading to correct for optical imperfections in the plate.

### 2.6 Multiplex ELISA

IL-6 and CXCL10 in **Supplementary Figures 2C–G** were detected using Multiplex ELISA from Meso Scale Discovery (MSD) U-plex platform according to manufacturer’s protocol (Meso Scale Diagnostics, Cat#: K15067L-2).

### 2.7 Western blot analysis

Samples were harvested in RIPA Lysis and Extraction Buffer (Thermo Scientific, Cat#: 89900) supplemented with protease and phosphatase Inhibitor (Millipore Sigma, Cat#: 11873580001 and Thermo Fisher Scientific, Cat#: A32961) and 10mM NaF (VWR, Cat#: J60251.AE). Before loading, the samples were mixed 1:1 with Laemmli buffer (Sigma Aldrich, Cat#: 38733) and boiled at 95°C for 5 min. The samples were loaded on a 10% Criterion™ TGX™ Precast Midi Protein Gel, 26 well (Bio-Rad Cat#: 567-1035) and run in MOPS Running Buffer (Thermo Fisher Scientific, Cat#: NP0001). The gel was blotted onto a Turbo Transfer Midi PVDF membrane (Bio-Rad Cat#: 170-4157) and the membrane was blocked with 5% skimmed milk (Sigma-Aldrich, Cat#: 70166). The membrane incubated overnight with primary antibody with rotation at 4°C, and hereafter incubated with secondary antibody for 1hour with rotation at room temperature before development with Clarity Western ECL Substrate (Bio-Rad, Cat#:1705060) or SuperSignal West Femto Maximum (Thermo Fisher Scientific, Cat#: 34095) using the Azure Biosystems imaging system 300. Primary antibodies were all used in ratio 1:1000 with 5% Bovine Serum Albumin (Roche, Cat#: 10735986001) except Vinculin, which was diluted 1:10000. Primary antibodies from Cell Signaling Technology: anti-TBK1 Cat#: 3313S (84 kDa), anti-STING Cat#: 13647S (33-42 kDa), anti-cGAS Cat#: 15102S (62 kDa), anti-IRF3 Cat#: 4302S (55 kDa), anti-pTBK1 Cat#: 5483S (84 kDa), anti-pSTING Cat#: 19781S (33-42 kDa), anti-STAT1 Cat#: 14994S (90 kDa), anti-pSTAT1, Cat#: 7649S (90 kDa). From Sigma Life Sciences: anti-Vinculin Cat#: V9131 (116 kDa) and from Abcam: anti-pIRF3 Cat#: 76493 (55 kDa). Secondary antibodies from Jackson ImmunoResearch: Peroxidase-AffiniPure F(ab’)2 Fragment Donkey Anti-Rabbit IgG Cat#: 711-036-152, Peroxidase-AffiniPure F(ab’)2 Fragment Donkey Anti-Mouse IgG Cat#: 715-036-150.

### 2.8 CRISPR mediated gene knockout

Genetically modified STING knock-out (KO) cell lines were generated using chemically modified guide RNA’s (sgRNAs) in combination with the nuclease, spCas9. Double strand breaks generated by spCas9 resulted in the creation of indels and ultimately knocking down the gene expression. sgRNAs were designed using the bioinformatic platforms CRISPick and CRISPOR and purchased from Synthego with a chemical 2’-O-methyl-3’phosphorothioate-modification on three terminal nucleotides in both ends. We designed two individual sgRNAs targeting approximately 50bp apart to increase the chances of a complete knockout of STING. Initially, ribonicleoprotein (RNP) complexes were formed by incubating 6μg spCas9 protein (Alt-R S.p. Cas9 Nuclease V3, Integrated DNA Technologies) with 3.2μg of each individual sgRNA for 15min. at room temperature. Hereafter, 100,000 cells were mixed with the RNPs and electroporated on the 4D-nucleofector (Lonza) in Nucleovette strips (Lonza) using the program CM138-P3. Cells were hereafter cultured under the same conditions as wildtype. sgRNA sequences provided in **Supplementary Table 2**.

### 2.9 RNA purification and cDNA synthesis

RNA was purified using RNeasy mini kit (Qiagen, Cat#: 74106) or NucleoSpin RNA kit (Macherey-nagel, Cat#: 740955) and eluted in 30-40μL RNase-free water. 250ng-1μg of RNA was used for cDNA synthesis using the iScript cDNA synthesis kit (Bio-Rad Cat#: 1708891BUN) on an Arktik thermal cycler (Thermo scientific) with program: 5’25˚C; 20’46˚C; 1’95˚C; 4˚C.

### 2.10 RT-qPCR

To determine expression levels of the human *IFNB1, IFNL1, IFNL2, IFNLR1 YWHAZ* and *EIF2B2* on lung cancer cell lines, gene specific primers were designed using Primer3 and purchased from Eurofins (**Supplementary Table 3**). Samples were analyzed in a final volume of 10μL, containing 5μL KAPA SYBR Fast Master Mix (Sigma-Aldrich, Cat#: KK4611), 2μM forward primer, 2μM reverse primer, nuclease-free water and 10ng of cDNA. Analysis was performed on a Lightcycler 480 platform with program: 3’95˚C; 40x (10”95˚C; 20”60˚C, 1”72˚C). Ct values were extracted using the Lightcycler Software. For obtaining gene expression values X0 was calculated as 2^-Ct^ and normalized to the X0 value of a reference gene. For each sample, two-three technical replicates were included and averaged during gene expression calculation.

### 2.11 RNA sequencing

Samples for RNA sequencing are generated in one experiment performed in duplicates. Cells were either left untreated or transfected with 2μg/mL of HT-DNA as described in the paragraph about transfection above. RNA was purified using RNeasy mini kit (Qiagen, Cat#: 74106) or NucleoSpin RNA kit (Macherey-nagel, Cat#: 740955) and eluted in 30-40μL RNase-free water.

RNA was sequenced either at BGI Copenhagen or Novogene Europe. A strand-specific and polyA-selected mRNA library was prepared, and the samples were sequenced at a depth of either 30 million reads per sample using paired-end reads of 100bp on a DNBSEQ platform (BGISEQ) or 20 million reads per sample using paired-end reads of 150bp on a Novaseq X plus platform.

The output fastq files were analyzed with R version 4.4.1 or the CLC Genomics Workbench 12 (QIAGEN) software. Using R or the RNA-seq module of the CLC Genomics Workbench 12, all reads were trimmed and mapped to the Genome Reference Consortium Human Build 38 (GRCh38/hg38). Expression values of each gene were normalized to transcripts per million (TPM).

### 2.12 Data analysis of RNA sequencing

TPM values from RNA sequencing were analyzed in the R software version 4.4.1. Initially all genes where all the samples had a TPM value < 5 were excluded. Hereafter a value of 1 was added to all TPM values to avoid zeros. A log2 fold change between the untreated and the HT-DNA transfected sample was calculated for each gene and each cell line. The average log2 fold change for each gene across the six cell lines was then calculated to rank the genes according to log2 fold change. The 50 genes with the highest log2 fold change were then included in a heatmap displaying the Z-score for each cell line, untreated (UT) and HT-DNA transfected (DNA).

### 2.13 Single Cell RNA sequencing data analysis

A comprehensive dataset of lung cancer single cell data from 316 patients, already processed and integrated (42), was downloaded from the Cell x Gene platform. We filtered the data on two parameters: 1) The samples should be either from lung cancer patients or healthy controls. 2) The samples were of lung tissue. The cell types were pre-annotated, and we simplified the cell type annotation as shown in **Supplementary Table 4**. The expression of IFNLR1 and IL10RB was visualized for both the healthy controls and cancer samples. For the visualization, the cells without expression for the two genes were removed. The difference in expression between the groups were evaluated by a Wilcoxon Rank Sum test calculated using the R package ggpubr. (Kassambara A (2023) ggpubr: ‘ggplot2’ Based Publication Ready Plots (R package version 0.6.0). https://CRAN.R-project.org/package=ggpubr).

### 2.14 Transcriptional activation of IFNLR1 using CRISPR activation

Transcriptional activation of IFNLR1 was carried out using chemically modified sgRNAs in combination with a mutant spCas9 nuclease with a defective nucleolytic activity (dCas9). Moreover, the dCas9 was fused to the three transcription activators p65, Rta, and VP64 (dCas9-VPR). sgRNAs were designed using the bioinformatic platforms CRISPick and purchased from Synthego with a chemical 2’-O-methyl-3’phosphorothioate-modification on three terminal nucleotides in both ends.

In total eight different sgRNAs targeting the promoter region of IFNLR1 were tested both alone and some in combination to determine which single sgRNA or combination of sgRNAs would result in the best transcriptional activation of IFNLR1. The dCas9-VPR mRNA was *in vitro* transcribed from a plasmid provided by Rasmus O. Bak (43).

For experiments including cells with transcriptionally activated IFNLR1, 1μg or each sgRNA was mixed with 0.5μg dCas9-VPR mRNA and added to 500.000 cells. A mock control with dCas9-VPR mRNA but no sgRNA is included in all experiments with CRISPRa. To deliver the CRISPRa components, cells were then electroporated on the 4D-nucleofector (Lonza) in Nucleovette strips (Lonza) using the program CM138-P3. sgRNA sequences provided in **Supplementary Table 5**.

### 2.15 Viability assay

For determining viability 5000 cells were seeded per well in a NUNC 96-well plate. 24 hours after seeding, the cells were treated with either IFNλ or IFNβ for 24 hours. Viability as percentage of an untreated control was determined using CellTiter 96 AQueous One Solution Cell Proliferation Assay (Promega, Cat#: G3580) according to manufacturer’s instructions.

### 2.16 Caspase 3/7 activation assay

Apoptosis resulting from caspase 3/7 activation after 48 hours treatment with approximated IC_50_ doses of cisplatin (Sigma-Aldrich, Cat#: 232120) and doxorubicin (Sigma-Aldrich, Cat#: D1515) was detected by flow cytometry. Cells were washed three times in PBS, trypsinized and resuspended in cell culture medium. Hereafter, cells were pelleted by centrifugation at 400xg for 5 minutes, resuspended in PBS and counted. Viability was determined from these cell counts. In each individual experiment, an equal number of cells were analyzed for each condition in a V-bottom 96-well plate (Sarstedt). Cells were resuspended in 95μL of PBS supplemented with 0.5% Bovine Serum Albumin (Roche, Cat#: 10735986001), 0.09% sodium azide (Sigma-Aldrich, Cat#: S2002), and 1mM EDTA (Invitrogen, Cat#: 15575020). To stain the cells for Caspase 3/7 activation, 5μL of Caspase-3/7 Reagent (Invitrogen, Cat#: R37111) were added to 95μL cell suspension and incubated in the dark for 30 minutes. Right before analyzing the samples, 2.5μL of 50μg/mL Propidium iodide Solution (Sigma-Aldrich, Cat#: P4864) were added to the samples. Cells were analyzed by flow cytometry measuring fluorescence emission at 530nm and 615nm using a NovoCyte Quanteon 4025 flow cytometer equipped with four lasers (405 nm, 488 nm, 561 nm and 637 nm) and 25 fluorescence detectors (Agilent, Santa Clara, CA). Data analysis was performed using FlowJo version 10.10.0. The gating strategy can be found in **Supplementary Figures 4**, **5**.

### 2.17 Statistics

The sample size and statistical methods used for each experiment have been provided in the respective figure legends. Data was analyzed with GraphPad Prism 10.2.3 (GraphPad Software) and R version 4.4.1. In cell experiments, comparison between groups was performed using Two-way ANOVA analysis using Šídák’s multiple comparisons test or multiple T tests with Šídák-Holm’s correction for multiple testing. For single cell RNA sequencing data, comparison of groups is done using Wilcoxon Rank Sum test. * = P <.05, ** = P <.01, *** = P value <.001, **** = P <.0001.

Figures were prepared using Biorender.com and PowerPoint.

## 3 Results

### 3.1 IFNλ is a broad marker of STING activation in NSCLC

**Figure 1:**
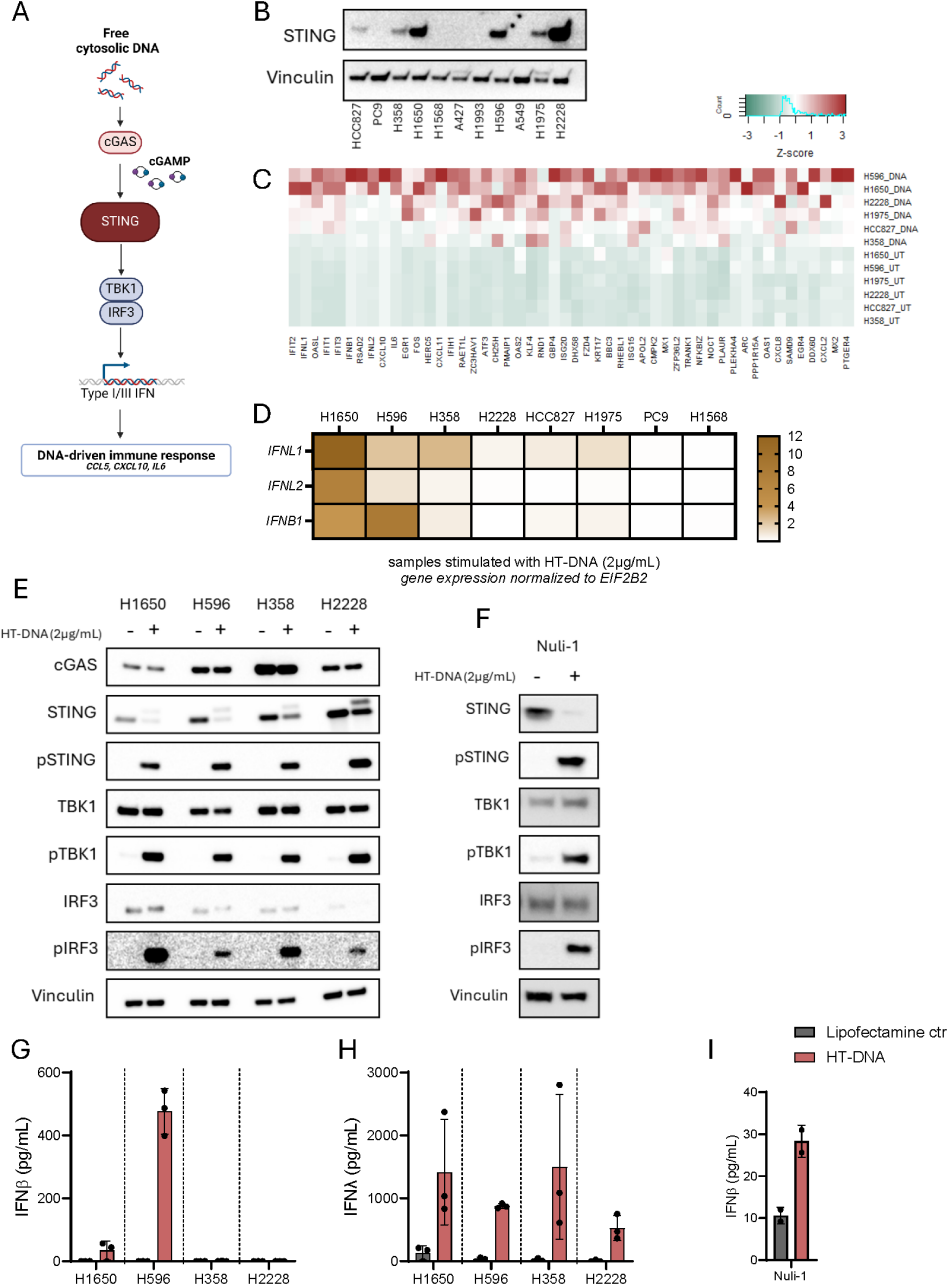
IFNλ is a broad marker of STING pathway activation in NSCLC. **(A)** An Illustration of the various factors essential for STING pathway activation. **(B)** Basal STING expression in 11 NSCLC cell lines detected using western blot. Vinculin was used as a loading control. **(C)** RNA sequencing data from six NSCLC cell lines demonstrating the expression levels of the 50 genes with the average highest log2 fold change, six hours after transfection with HT-DNA (2μg/mL). Data presented as heatmap with Z-scores. **(D)** A heatmap of *IFNL1*, *IFNL2*, and *IFNB1* gene expression six hours post stimulation with HT-DNA (2ug/ml), measured by RT-qPCR, in eight NSCLC cell lines. **(E, F)** Evaluation of STING pathway activation in four NSCLC cell lines and the epithelial control cell line, Nuli-1, following transfection with HT-DNA (2μg/mL) or control for six hours. The expression of total STING, phospho(Ser366)STING, total TBK1, phospho(Ser172)TBK1, total IRF3, and phospho(Ser386)IRF3 were evaluated by western blot using vinculin as loading control. **(G-I)** NSCLC cell lines and Nuli-1 were stimulated with HT-DNA (2μg/mL) formulated with lipofectamine 2000. Supernatants were harvested after 20 hours and analyzed for IFNβ and IFNλ expression by ELISA. In **(G, H)** data is shown as mean +/- standard deviation of three independent experiments conducted in triplicates. In **(I)** mean of one experiment performed in duplicates is shown with +/- standard deviation indicated.

As STING protein expression is a prerequisite for pathway activity (**Figure 1A**), we conducted an initial screening of STING expression in 11 different NSCLC cell lines. This revealed that approximately half of the cell lines did not express STING while the cell lines HCC827, H358, H1650, H596, H1975, and H2228 did (**Figure 1B**). We next evaluated the functionality of STING in each of the cell lines by measuring type I IFN and IL-6 in response to exogenous DNA stimulation, in form of lipofectamine delivered herring-testis (HT)-DNA. As control, we included the TLR3 agonist, Poly I:C, which induces secretion of the measured cytokines by relying on the same transcription factors (**Supplementary Figures 1A–D**). We were only able to detect type I IFN from four of the six STING-expressing cell lines (H1650, H596, H358 and to a lesser extent in H1975) in response to HT-DNA (**Supplementary Figure 1A**). These and additional two cell lines did also produce type I IFN in response to Poly I:C (**Supplementary Figure 1B**). All STING-expressing cell lines produced IL-6 (to various degrees) in response to HT-DNA stimulation, but interestingly, so did two of the non-STING expressing cell lines (PC9 and H1568; **Supplementary Figure 1C**). All, with exception of two cell lines (A427 and A549), did secret IL-6 in response to Poly I:C (**Supplementary Figure 1D**). These results suggested that type I IFN is a poor marker for STING pathway activation in NSCLC.

We next investigated the transcriptomic profile induced by HT-DNA in the six STING-expressing NSCLC cell lines by RNA sequencing. A waterfall plot of average log2 fold change across all cell lines showed that DNA stimulation both decreased and increased gene expression although the increase led to more genes with a higher log2 fold change (**Supplementary Figure 1E**). In fact, 87 genes had an average log2 fold change above 2 whereas 0 genes were found to have an average log2 fold change below -2 (**Supplementary Table 1**). An inspection of the 50 most upregulated genes across the cell lines showed a classical IFN-signature with expression of ISGs like IFIT2, IFIT3, RSAD2, IFIT1, OASL, ISG15, PLAUR and KLF4. Interestingly, the average upregulation of the less studied type III IFNs, IFNL1 and IFNL2, were both in the top 10 of upregulated genes together with IFNB1. It was also apparent that interferon expression in general was very cell type dependent with H2228 not upregulating IFNB1 at all and H1975, HCC827, and H358 only to a lesser extent in response to HT-DNA stimulation. In terms of IFNL1, it was found to be upregulated to a higher degree across all cell lines (**Figure 1C**). This observation was confirmed when we compared expressions of IFNL1, IFNL2 and IFNB1 across eight NSCLC cell lines after HT-DNA stimulation. Here IFNL1 was relatively higher expressed compared to IFNB1, with exception of H596. For the two STING-negative cell lines PC9 and H1568 both type I and III IFNs were undetectable (**Figure 1D**).

Next, we conducted a deeper characterization of four STING expressing cancer cell lines and compared them to the immortalized healthy epithelial cell line Nuli-1. All cancer cell lines as well as Nuli-1 showed phosphorylation of STING, TBK1, and IRF3 after stimulation with HT-DNA, indicating an active signaling pathway (**Figures 1E, F**). However, H596, H1650 and Nuli-1 were the only cell lines producing IFNβ in response to HT-DNA stimulation (**Figures 1G–I**). Interestingly, IFNβ could be detected upon Poly I:C stimulation in H358 but not at all in H2228 (**Supplementary Figure 2A**). All four cancer cell lines had detectable IFNλ levels upon HT-DNA as well as Poly I:C stimulation (**Figure 1H**, **Supplementary Figure 2B**). Additionally, all cell lines also produced IL-6 and CCL5 in response to HT-DNA, whereas H1650 and H2228 were not able to produce CXCL10 (**Supplementary Figures 2C–G**).

Taken together, these results suggests that IFNλ could be a more relevant marker for an active STING pathway in NSCLC compared to type I IFN and CXCL10.

### 3.2 IFNλ and IFNβ share similar cell type dependent kinetic patterns

**Figure 2:**
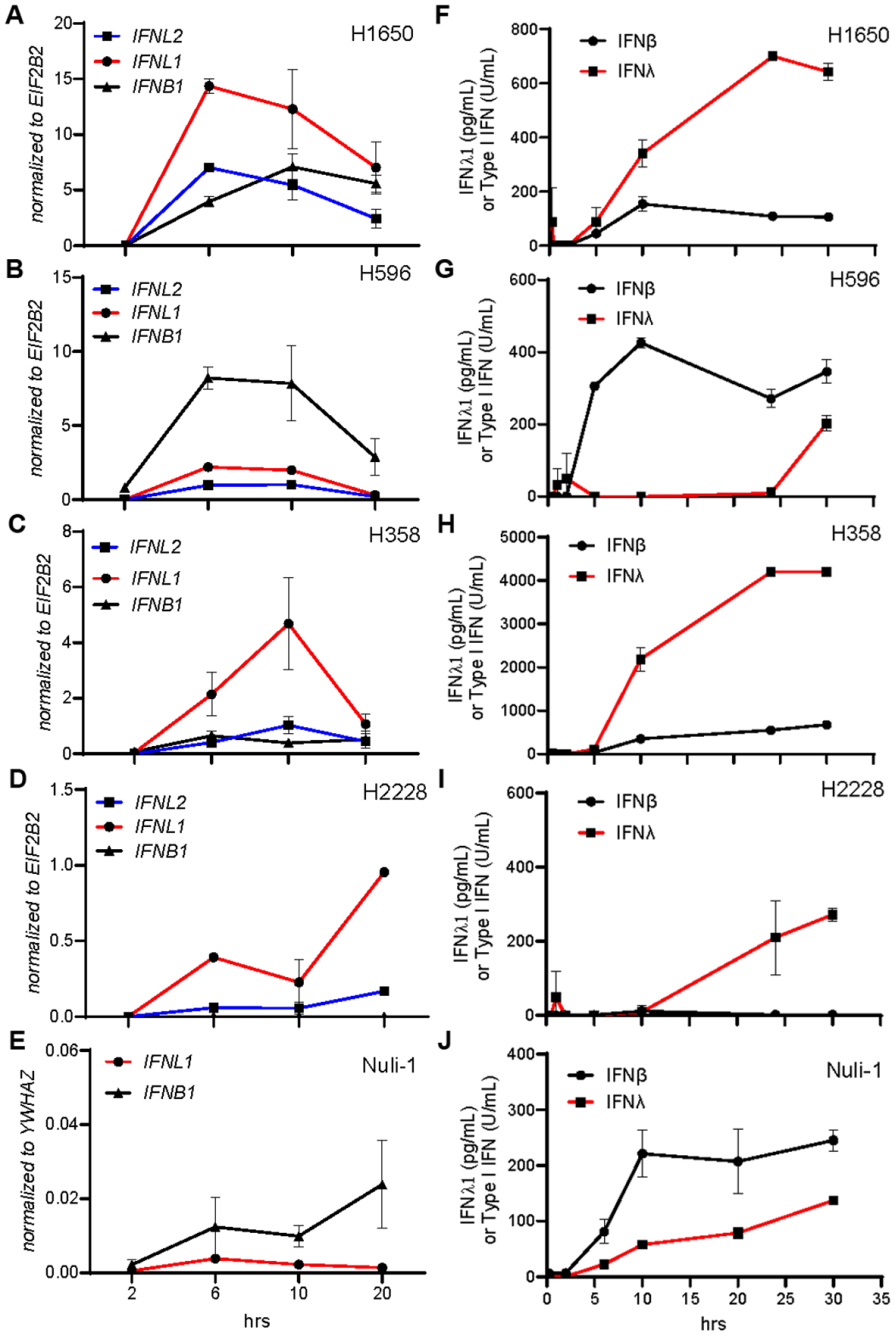
IFNλ and IFNβ share similar cell type dependent kinetic patterns. NSCLC cell lines and Nuli-1 cells were treated with HT-DNA (2μg/mL) formulated with lipofectamine 2000, for the indicated number of hours (hrs), where after gene **(A-E)** and protein **(F-J)** expression levels of *IFNB1*, *IFNL1*, and *IFNL2* were determined. Data is shown as mean +/- standard deviation of duplicates from one experiment.

Since we found the different cancer cell lines to generally secrete more type III IFNs than type I IFN, we wanted to determine if this correlated to alternative gene expression kinetics. Thus, we stimulated each cancer cell line with HT-DNA and measured expression of IFNL1, IFNL2, and IFNB1 after 2, 6, 10 and 20 hours (**Figures 2A–E**). In accordance with our earlier findings, we observed that IFNL1 was relatively higher expressed than IFNB1 in three of the four NSCLC cell lines and followed an expression kinetic pattern like IFNB1 (**Figures 2A–D**). The cell line H596 and Nuli-1 did stand out having higher IFNB1 expression (**Figures 2B, E**). These observations were mirrored in the protein secretion pattern (**Figures 2F–J**).

### 3.3 Doxorubicin and cisplatin induce IFNL1 and IFNB1 transcription

**Figure 3:**
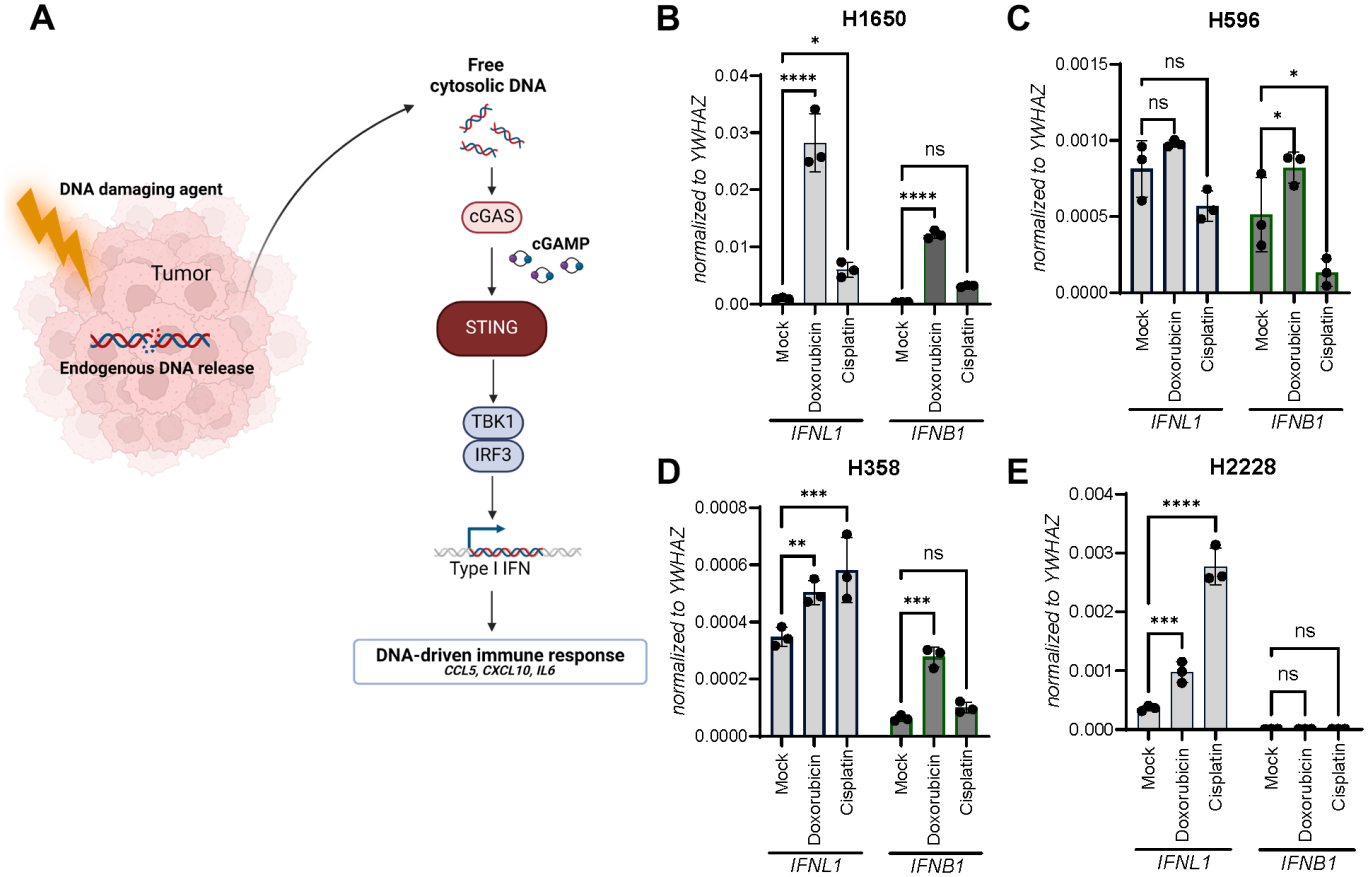
Doxorubicin and cisplatin induce *IFNL1* and *IFNB1* transcription. **(A)** An illustration of chemotherapy-induced STING pathway activation. **(B-E)** Gene expression of *IFNL1* and *IFNB1* was determined in NSCLC cell lines after a single IC_50_-value treatment with doxorubicin or cisplatin for 48 hours. Data is shown as mean +/- standard deviation of triplicates. Two-way ANOVA analysis using Šídák’s multiple comparisons test is performed. *P <.05, **P <.01, ***P value <.001, ****P <.0001.

Sensing of endogenous DNA in cancer cells through the cGAS-STING pathway facilitated by DNA-damaging agents has been proposed to initiate anti-tumoral responses (**Figure 3A**). From our initial characterization of the four NSCLC cell lines, we observed a diverse pattern of cGAS protein levels with H358 expressing the most and H1650 the least cGAS (**Figure 1E**).

To determine how IFNL1 and IFNB1 expression was regulated upon DNA damage-inducing agents, each cell line was treated with doxorubicin or cisplatin for 48 hours. Interestingly, IFNL1 expression was significantly upregulated by both doxorubicin and cisplatin in three of the cancer cell lines and did not seem to correlate with cGAS protein expression (**Figures 3B–E**). Once again H596 stood out being the only cell line demonstrating significant upregulation of only IFNB1 and not IFNL1 expression (**Figure 3C**).

**Figure 4:**
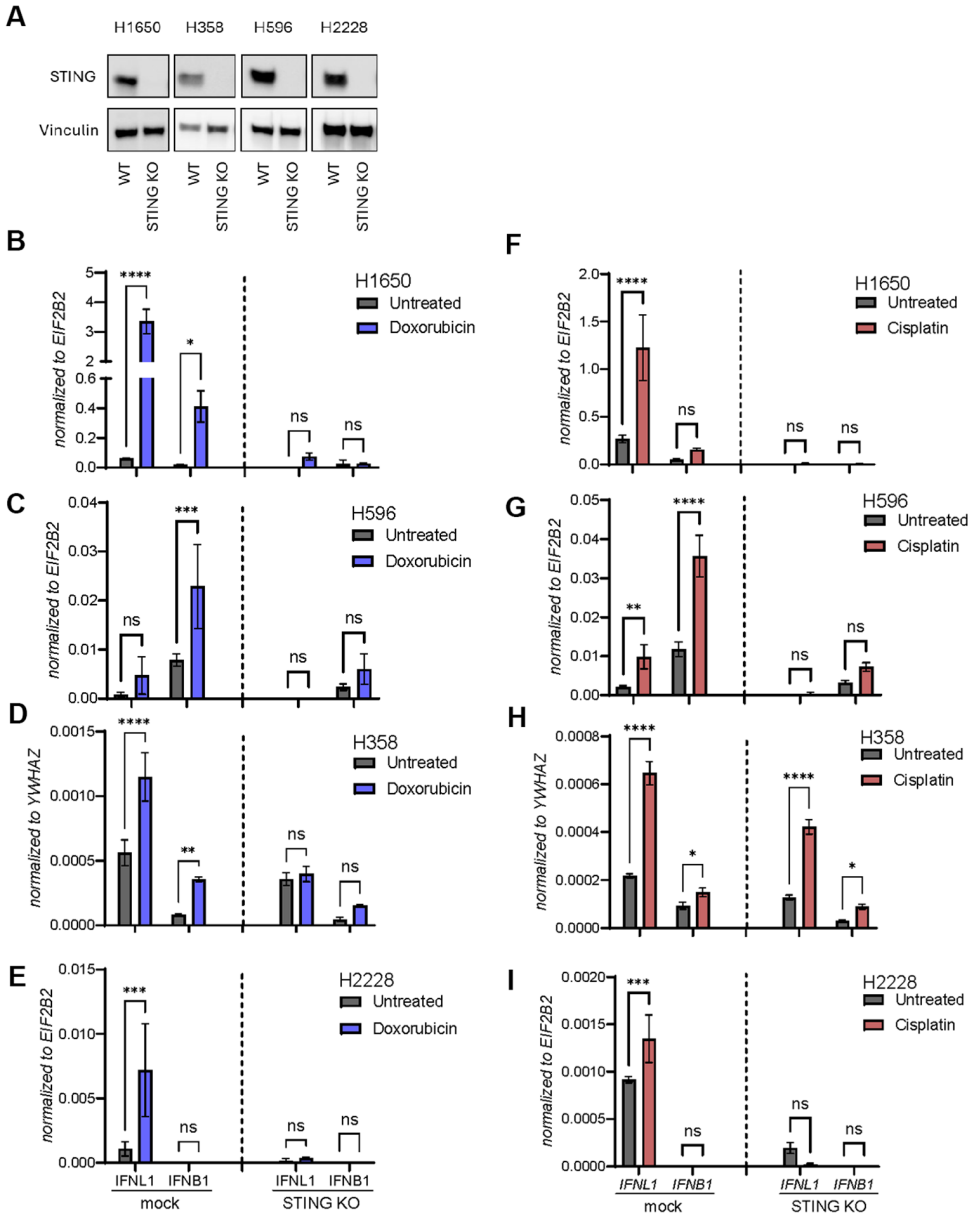
Doxorubicin and cisplatin induce *IFNL1* and *IFNB1* transcription in a STING-dependent manner. **(A)** Western blot of STING expression measured in wild type (WT) or STING knockout (KO) of four NSCLC cell lines. Vinculin is used as loading control. **(B-I)** Mock and STING KO NSCLC cell lines were stimulated with doxorubicin **(B-E)** or cisplatin **(F-I)** for 48 hours. Cells were then harvested and gene expression of *IFNL1* and *IFNB1* determined by RT-qPCR. Data is shown as mean +/- standard deviation of triplicates. Two-way ANOVA analysis using Šídák’s multiple comparisons test is performed. *P <.05, **P <.01, ***P value <.001, ****P <.0001, ns = none significant.

To confirm that responses were truly STING-dependent, we next used CRISPR-Cas9 technology to generate a STING knockout (KO) variant of each NSCLC cell line. The level of STING depletion was confirmed by western blot (**Figure 4A**). Next, a control KO (mock) and STING KO cells were treated with doxorubicin (**Figures 4B–E**) or cisplatin (**Figures 4F–I**) for 48 hours followed by expression analysis of IFNB1 and IFNL1. Overall, these results confirmed that the DNA damage-inducing agents triggered IFNB1 and IFNL1 expression in a STING-dependent manner.

### 3.4 IFNLR1 expression is cell type dependent and downregulated in tumor cells

**Figure 5:**
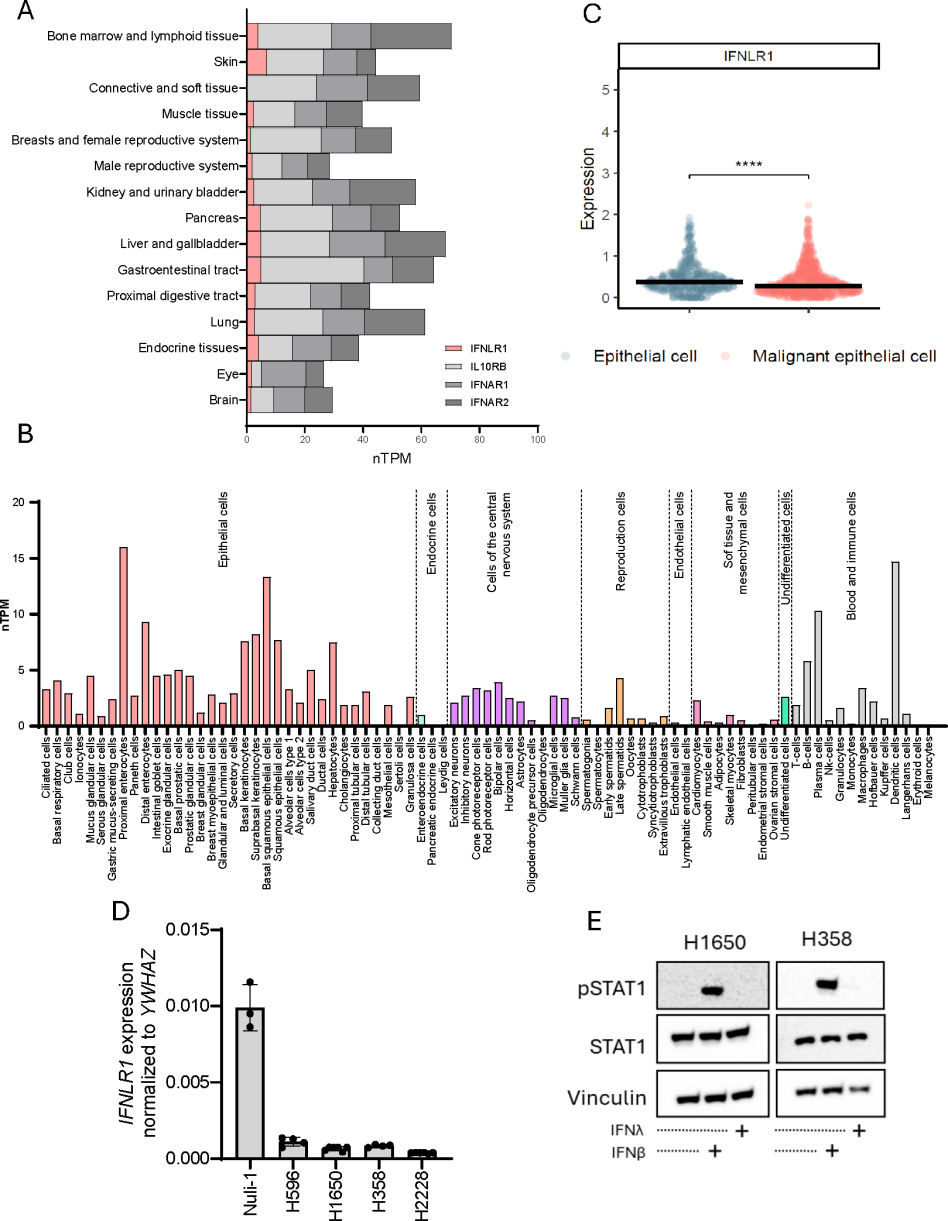
*IFNLR1* expression is cell type dependent and downregulated in tumor cells. **(A)** Gene expression as normalized transcripts per million (nTMP) of *IFNLR1*, *IL10RB*, *IFNAR1*, and *IFNAR2* expression in different human tissues generated from the Human Protein Atlas (proteinatlas.org) (59). **(B)** *IFNLR1* gene expression as nTMP in different tissues generated from the Human Protein Atlas (proteinatlas.org) (65). **(C)** The expression of *IFNLR1* in healthy and malignant epithelial cells obtained from 316 patients (healthy controls or lung cancer patients) using single cell RNA sequencing (42). The difference in expression between the groups was evaluated by a Wilcoxon Rank Sum test. ****P <.0001. **(D)** The basal gene expression of *IFNLR1* in four NSCLC cell lines and Nuli-1 cells measured by RT-qPCR. Values are shown as mean +/- standard deviation from 3-5 replicates from one or two experiments. **(E)** Two NSCLC cell lines were treated for 15 min. with either IFNλ (100ng/mL), IFNβ (100ng/mL) or left untreated prior to cell lysis. The protein expression of STAT1 and pSTAT1 was detected by western blot. Vinculin was used as loading control.

A major difference between type I and type III IFNs is how they modulate a local environment through autocrine and paracrine receptor interactions. We used the human protein atlas database to examine the expression pattern of IFN receptor subunits. The type I IFN receptor subunits, IFNAR1 and IFNAR2, as well as type III IFN receptor subunit IL-10Rβ are ubiquitously expressed in our body whereas the type III IFN receptor subunit IFNLR1 is expressed at low levels and more selectively specific organs (**Figure 5A**). Furthermore, exploration of numerous cell types demonstrated that IFNLR1 expression is very sparce and clearly cell type specific (**Figure 5B**). Next, using a publicly available single cell RNA sequencing dataset from lung cancer tumors (42), we found IFNLR1 expression to be significantly decreased in malignant epithelial cells compared to normal epithelial cells from the same tumor (**Figure 5C**). Furthermore, we could demonstrate that IFNLR1 expression was lower in our studied NSCLC cell lines compared to Nuli-1 (**Figure 5D**), suggesting that IFNLR1 is selectively downregulated in NSCLC both in patient tumors, and in *in vitro* cell line models. Next, we wanted to explore the ability of IFNλ and IFNβ to induce an IFN-mediated response in our NSCLC cell lines. Knowing that IFNλ and IFNβ activates the same downstream signaling pathway after receptor engagement involving phosphorylation of STAT1 we stimulated two of our NSCLC cell lines with recombinant IFNλ and IFNβ to evaluate STAT1 phosphorylation. Here, we only identified phosphorylation of STAT1 in response to IFNβ (**Figure 5E**).

The selective downregulation of IFNLR1 expression in cancer cells could be seen as an immune escape mechanism evolved during cancer development which allows cancer cells to secrete IFNλ to support immunomodulation of the tumor microenvironment while avoiding being affected themselves.

### 3.5 Transcriptional upregulation of IFNLR1 increases IFNλ sensitivity and leads to decreased viability

**Figure 6:**
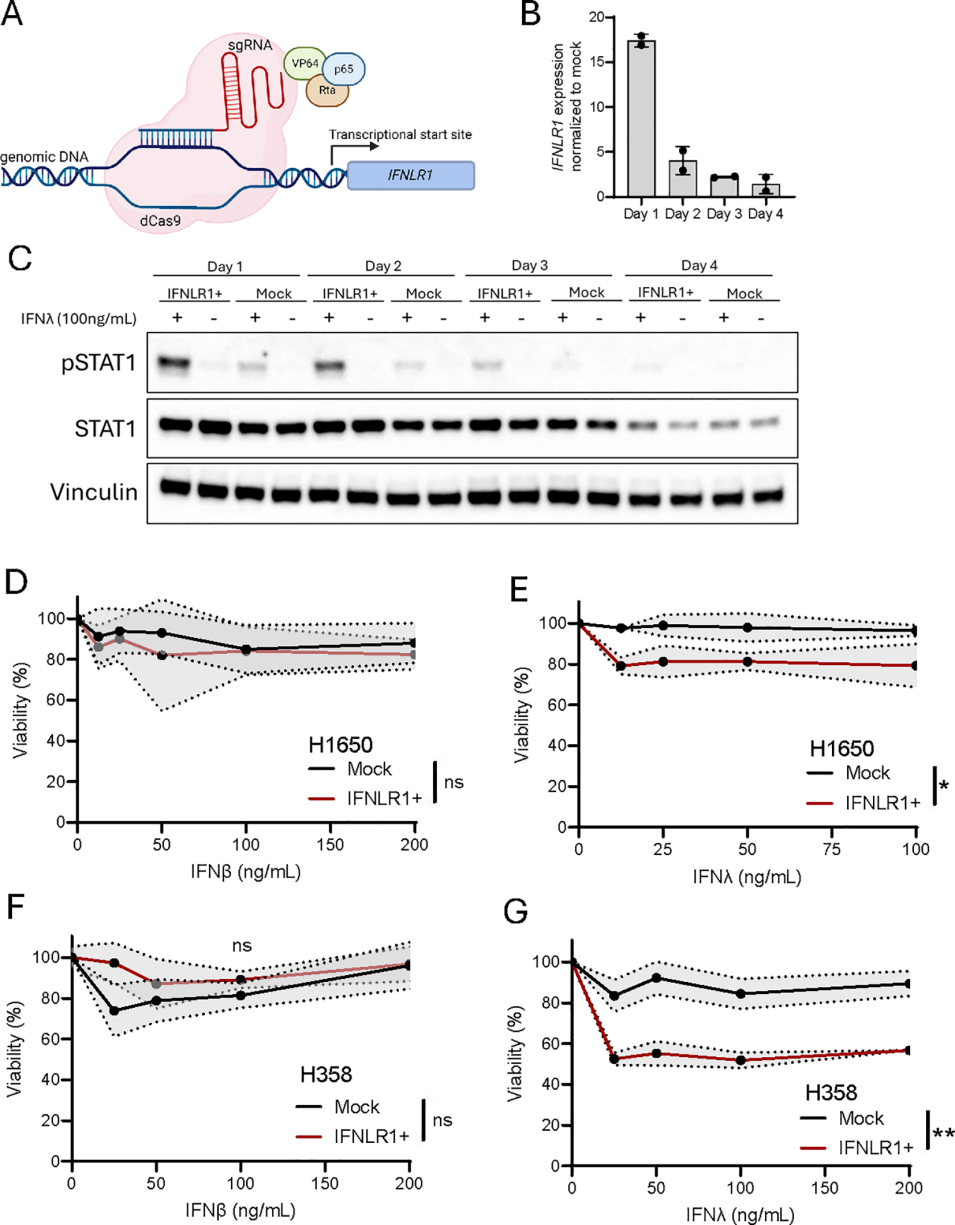
Transcriptional upregulation of *IFNLR1* increases IFNλ sensitivity and leads to decreased viability. **(A)** Illustration of CRISPR activation (CRISPRa) of *IFNLR1*. Created using Biorender.com. **(B)** The H1650 cell line was subjected to CRISPRa of *IFNLR1*, and gene expression evaluated at day 1-4 after treatment. The *IFNLR1* expression was normalized to H1650 mock control cells in which transcriptional activation was not performed. The data is shown as mean +/- standard deviation of two replicates from a single experiment. **(C)** The H1650 cell line was subjected to CRISPRa of *IFNLR1* (IFNLR1+). At the indicated days, cells were treated with IFNλ (100ng/mL) or left untreated for 15 min. prior to harvest. The level of STAT1 and pSTAT1 expression was determined by western blot. H1650 mock control cells in which transcriptional activation was not performed were included for each time point. Vinculin was used as a loading control. **(D-G)** Viability measurement of H1650 **(D, E)** and H358 **(F, G)** cell lines. One day after CRISPRa of *IFNLR1* and seeding, cells were treated with the indicated concentrations of IFNλ or IFNβ for 48 hours. Viability was compared to an untreated control for both IFNLR1+ and mock cells. Data is shown as mean +/- standard deviation (shaded area) of the average of four replicates from two-three experiments in **(D-E, G)** In **(F)** mean +/- standard deviation of four replicates are shown. Two-way ANOVA analysis using Šídák’s multiple comparisons test is performed comparing mock and IFNLR1+ in samples treated with 100ng/mL of IFNβ or IFNλ. *P <.05, **P <.01, ns = none significant.

Gene regulation can be achieved by various epigenetic drugs and is already used in various cancer settings (44). However, most drugs are often not specific to a single gene of interest but affects multiple genes within a single cell. Thus, to investigate the specific role of IFNLR1 expression and pathway activation in NSCLC, we established a CRISPR-mediated transcriptional activation approach (CRISPRa) (**Figure 6A**) targeting the promotor region of IFNLR1. We combined mRNA of dead Cas9-VPR with a total of eight different guide RNAs (sgRNAs) which we tested individually and in combination to identify the best sgRNA(s) for transcriptional activation following electroporation of the RNA molecules (**Supplementary Figure 3A**). Ultimately a combination of two gRNAs resulted in a clear upregulation of IFNLR1 and was associated with robust receptor signaling measured by phosphorylation of STAT1 (pSTAT1) (**Supplementary Figure 3B**). Next, we evaluated the kinetics of IFNLR1 mRNA expression and pSTAT1 signaling following CRISPRa in H1650 cells. The gene expression of IFNLR1 peaked one day after electroporation and was barely detectable after four days (**Figure 6B**). Regarding receptor activation in the cells, we observed pSTAT1 expressed up to three days after stimulation with IFNλ (**Figure 6C**).

It is well known that interferon signaling can affect cancer cell viability negatively (37–39, 45–47). To test this in our settings, we first treated cells with recombinant IFNβ as we already knew that the cancer cell lines were sensitive to IFNβ signaling (**Figure 5E**). This resulted in a minimal decrease in cell viability of 10-20% compared to untreated cells (**Figures 6D, F**) but not in a dose-dependent manner. When treating with recombinant IFNλ, both cancer cell lines were unaffected by the treatment but upon CRISPRa of IFNLR1 we observed a significant drop in cell viability of 20-40% (**Figures 6E, G**). These results encouraged us to investigate the potential of transcriptionally upregulating IFNLR1 as a way of sensitizing NSCLC cells to endogenous IFNλ secretion.

### 3.6 Transcriptional upregulation of IFNLR1 supports chemotherapy-induced apoptosis

**Figure 7:**
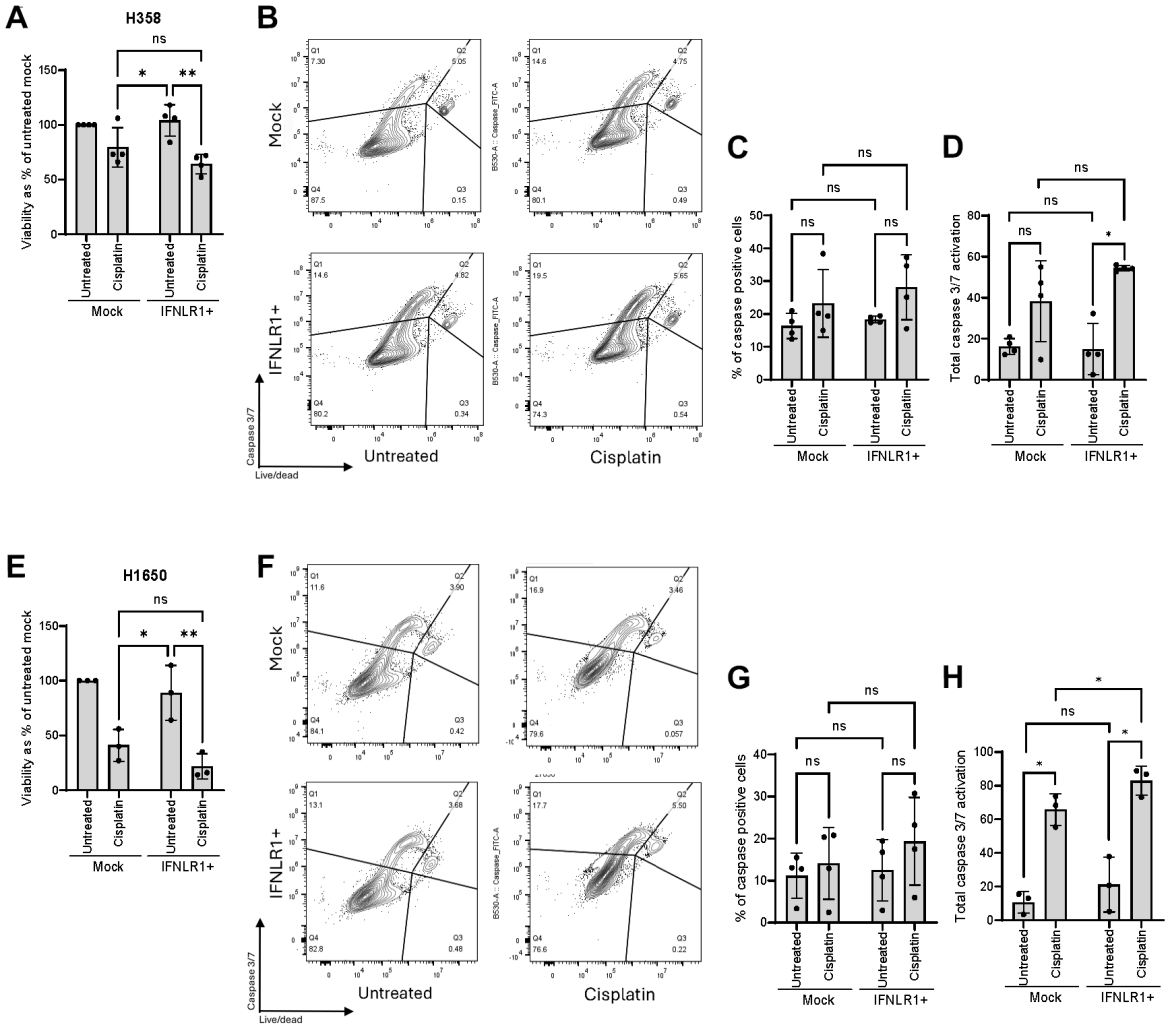
Transcriptional upregulation of IFNLR1 supports chemotherapy-induced apoptosis. Two NSCLC cell lines were treated with cisplatin for 48 hours with (IFNLR1+) or without (Mock) CRISPRa for IFNLR1. **(A, E)** Viability was measured by cell count and normalized as percentage of an untreated mock. **(B, C, F, G)** Caspase 3/7 activation as an early marker of apoptosis was determined by flow cytometry. **(B, F)** show representative gating strategies. For data in **(A, C, E, G)**, mean +/- standard deviation of 3-4 individual experiments are shown. Two-way ANOVA analysis using Šídák’s multiple comparisons test is performed. *P <.05, **P <.01. **(D, H)** A score of total caspase 3/7 activation combining both cells dead prior to flow cytometry analysis and cells analyzed by flow cytometry was calculated. Mean +/- standard deviation of 3-4 individual experiments are shown. Multiple paired T tests using Holm-Šídák’s method for multiple testing was performed. *P <.05, **P <.01, ns = none significant.

Cisplatin induces inter- and intrastrand cross-links (48), and moreover contributes to cellular disfigurement from oxidative stress and DNA damage. We also found that cisplatin treatment of our NSCLC cell lines induced measurable IFNλ levels (**Figures 3B–E**). Thus, to evaluate if chemotherapy-induced type III IFN production was enough to trigger IFNLR1-mediated apoptosis, we finally performed transcriptional upregulation of IFNLR1 (IFNLR1+) in combination with cisplatin treatment for 48 hours. In our two NSCLC cell lines, we observed that cisplatin treatment resulted in a decreased viability measured by cell count and this seemed to be slightly increased in IFNLR1+ cells (**Figures 7A, E**). Next, we used the more appropriate caspase 3/7 activation assay as an early marker of apoptosis which we evaluated by flow cytometry (gating strategy in **Supplementary Figures 4**, **5**). Here, we observed a notable change in the caspase 3/7 activation in both cell lines, however not statistically significant (**Figures 7B, C, F, G**). To capture both the effect of cells undergoing apoptosis and those that had already died at the time of our analysis after 48 hours of cisplatin treatment, we introduced a total dead-caspase 3/7 activation score. The score combined the percentage of caspase positive cells with percentage of dead cells calculated with the assumption that 100% of the dead cells were positive for caspase 3/7 activation during apoptosis. In IFNLR1+ conditions, we observed significantly increased total caspase 3/7 activation score when cells had been exposed to cisplatin treatment compared to mock in both cell lines (**Figures 7D, H**). In H1650, we also observed significantly higher total caspase 3/7 activation in cisplatin-treated IFNLR1+ cells compared to cisplatin-treated mock cells. Overall, these results indicate that transcriptional activation of IFNLR1 can sensitize NSCLC cell lines to cisplatin and potentiate cisplatin-induced apoptosis.

## 4 Discussion

The cGAS-STING pathway has been highlighted as a central immunology pathway in mounting antitumoral responses in the TME. Sensing of cytosolic DNA resulting from DNA damage and chromosomal instability and secretion of inflammatory cytokines makes the TME immunological “hot” and prone for better adaptive immune responses (11, 13, 49). While pathway activation is typically assessed by expression of inflammatory cytokines such as CCL5, type I IFNs, and CXCL10 (16, 19), it can also trigger the release of other cytokines depending on the tissue type. Here, we found that NSCLC cell lines produced type III IFNs and only limited type I IFN, in response to stimulation with HT-DNA and the DNA damaging agents, doxorubicin and cisplatin. Importantly, we found that this response was STING-dependent across all cancer cell lines. The mechanisms of cytosolic DNA being detected by cGAS and leading to downstream STING, TBK1, and IRF activation and ultimately resulting in the release of type I IFN is well established. Apart from activation of IRF3 the transcription factor nuclear factor kappa B (NF-κB) can also be activated in this process leading to the release of inflammatory cytokines (14). The exact DNA sensing mechanism(s) leading to the release of STING-dependent type III IFN in currently unknown, however both cGAS, Ku70, and DNA-PK have been proposed (31, 32, 50, 51). Downstream of STING, type III IFN gene induction have been proposed to differ from that of type I IFN by involving IRF1 and IRF7 as well as IRF3 (31). However, further research should be done to establish the exact mechanism involved in STING-mediated type III IFN release.

Interruption of type I IFNs production in cancer is a known immune evasion mechanism preventing a systemic anti-tumor immune response. However, only a few immune cell subsets, including dendritic cells, B cells, and macrophages, express IFNLR1 making them responsive to type III IFNs (**Figure 5B**). Investigations on how IFNλ affects the antitumoral immune response is currently limited. In a recent preclinical study, ectopic IFNλ expression by tumor cells increased immune cell infiltration associated with antitumoral effects (52). Furthermore, this study also suggested that high IFNλ3 expression in bladder cancer correlates with immune cell infiltration and efficacy of immunotherapy. Another recent study suggested that engagement of the STING pathway using antibody-drug-conjugates in tumor settings supports IFNλ expression and anti-tumor activities (53). These studies together with our data indicate that we should widen our view on the role of IFNs and look beyond the sole effect of type I IFNs.

It is notable to observe that the receptor system responding to type III IFNs is merely expressed in benign but not malignant epithelial cells. We can only speculate why cancer cells may evolve in a manner that silences this receptor; however, we hypothesize that it may be an immunological escape mechanism. In our work, we found that this could be targeted by upregulating IFNLR1 and thereby sensitizing the cancer cells to IFNλ leading to reduced viability. Supporting this hypothesis, a TCGA analysis across tumor types reported a superior relapse-free survival for patients expressing high levels of IFNLR1 (54). Whether this is driven by expression within the tumor cells, or the immune cells remains to be elucidated.

The parallels between type III IFN and type I IFN signaling imply that known benefits of type I IFNs in cancer treatment may extend to type III IFNs. Trials of high-dose IFN adjuvant therapy in high-risk patients with melanoma (stage IIb or stage III disease) shows an extension of relapse-free and overall survival and highlights IFN as a valid therapeutic option (55–57). However, one of the largest obstacles to the use of type I IFNs is the dose-limiting adverse effects, including influenza-like symptoms, nausea, depression and leukopenia (57). The tissue-dependent expression of IFNLR1, particularly in epithelial cells, raises an interesting question – will the selective specificity make IFNλ a better alternative for treating cancer patients with NSCLC and other cancer with epithelial origin? A comparative study investigating treatment of hepatitis B patients with peginterferon lambda-1a or peginterferon alpha-2a revealed distinct adverse effects between the two interferon subtypes (58). Interestingly, peginterferon lambda-1a led to markedly fewer cases of influenza-like symptoms, leukopenia and dose-reductions than peginterferon alpha-2a, though it did cause more hepatobiliary events consistent with IFNLR1 expression in hepatocytes and liver endothelial cells (59). Additionally, a recent phase III clinical trial of pegylated IFNλ administered to patients with COVID-19 showed good tolerance and strong antiviral effects (60). These findings combined suggest a potential cancer therapy approach targeting type III IFN signaling to obtain better tolerance and less adverse effects compared to type I IFN-based treatments. Apart from improved tolerance, it would be of great relevance to investigate the role if IFNλ-signaling in reducing tumor growth. Attempts with generating different murine cancer cell lines stable expressing IFNλ and subsequent engraftment in IFNLR1 wildtype mice shows tumor clearance (40, 52, 61). Also, a few IFNLR1 knockout mouse models exist, but has primarily been studied in context of viral infections (62–64). To our knowledge no *in vivo* studies has been carried out comparing the tumor growth in an IFNLR1-deficient mouse model of epithelial origin or the use of recombinant Pegulated-IFNλ to evaluate the potential antitumorigenic role of drug-targeting the IFNλ-signaling pathway. Combing the results from our work with the few existing cancer relevant papers, do support the future importance of exploring the role of selective type III interferon therapy in cancer settings.

Our investigation into combining transcriptional activation of IFNLR1 and chemotherapy induced IFNλ signaling may be a promising axis to potentiate antitumoral responses. Further investigations should be conducted including optimization of dose and timing to strengthen this hypothesis. Based on the growing number of trials combining STING pathway agonist with especially immune checkpoint inhibitors (NCT04609579, NCT04147234, NCT04167137, NCT04096638, NCT03956680, NCT03937141) but also radiotherapy (NCT02379845) and chemotherapy (NCT00674102, NCT00832494, NCT01057342), it will become interesting to further elucidate the role of STING pathway mediated type III IFN as an active player in the effects of those combinations. With improvement of mRNA therapeutics and lipid nanoparticle delivery, we may in the future explore if selective upregulation of IFNLR1 in cancer cells support better overall survival when combined with traditional cancer therapies.

In summary, our findings demonstrate that activation of the STING pathway in NSCLC is more prone to induce type III IFNs than type I IFNs, and thus we need to expand our focus on how different interferons may contribute to modulate the tumor microenvironment. Perhaps type III IFN is a better proxy for STING activation in the TME and biomarker for effect of immunotherapies. Finally, understanding how and why cancer cells silence the expression of IFNLR1 may give us novel cancer drug targets for the future.

## Data Availability

The original contributions presented in the study are publicly available. This data can be found here: Gene Expression Omnibus (GEO) repository, accession number GSE288796.
