# Supplementary material for: IFNλ1 is a STING-dependent mediator of DNA damage and induces immune activation in lung cancer
Source: Front Immunol. 2025 Feb 12;15:1525083. doi: 10.3389/fimmu.2024.1525083 (PMC11862833; doi:10.3389/fimmu.2024.1525083)
Supplement: Supplementary file 1 [file DataSheet1.docx]

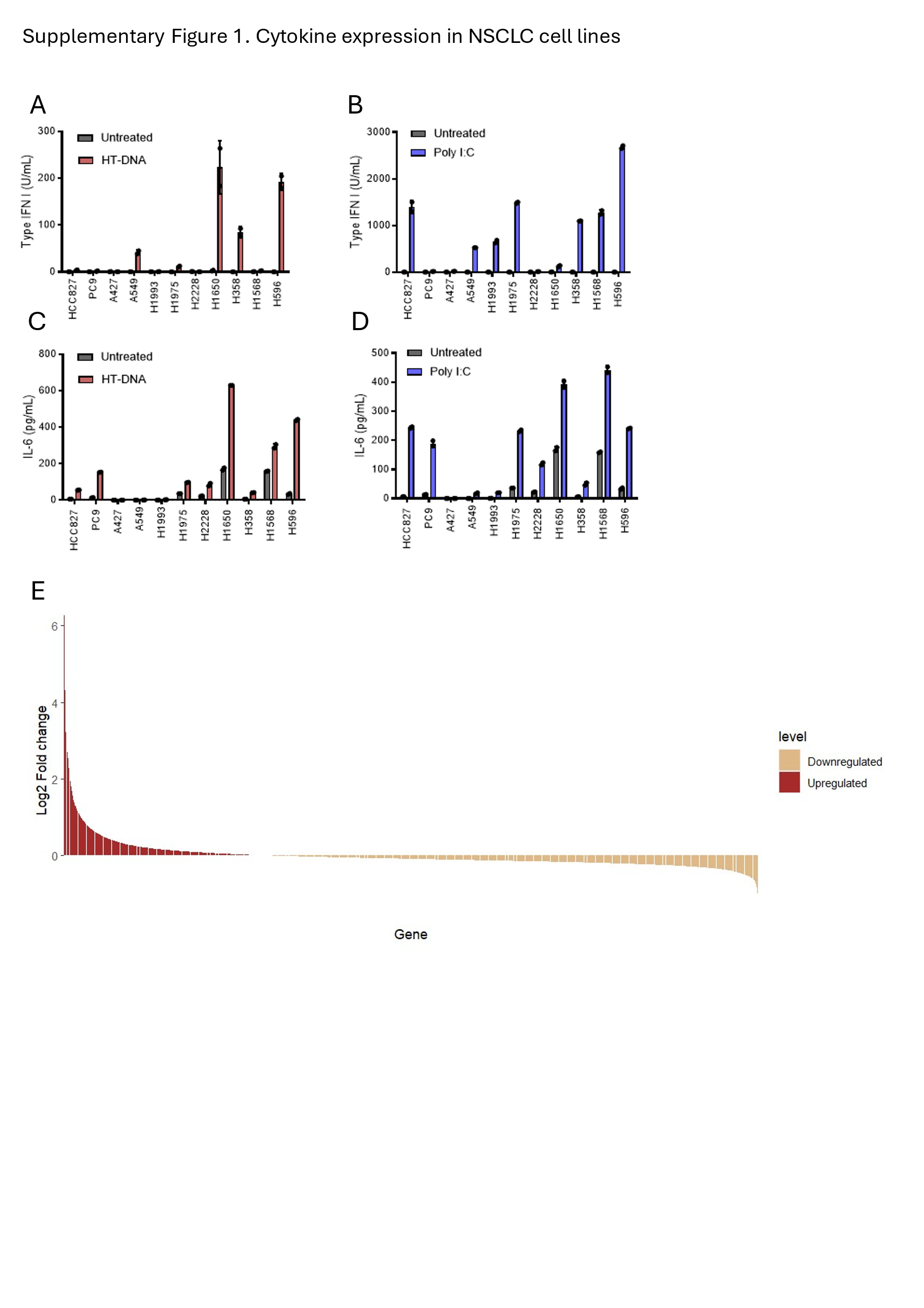


**Supplementary Figure 1. Cytokine expression in NSCLC cell lines.** (**A-D**) Type I IFN and IL-6 expression in supernatants from 11 NSCLC cell lines. Type I IFN is measured using a biofunctional reporter cell assay and IL-6 is measured using ELISA. Cells are transfected for 20 hours with HT-DNA (2μg/mL), Poly I:C (40ng/mL), or a control. Mean +/- standard deviation of duplicates from one representative experiment out of three is shown. (**E**) Waterfall plot depicting the average log2 fold change across six NSCLC cell lines six hours after transfection with HT-DNA (2μg/mL). Gene expression was measured using RNA sequencing.


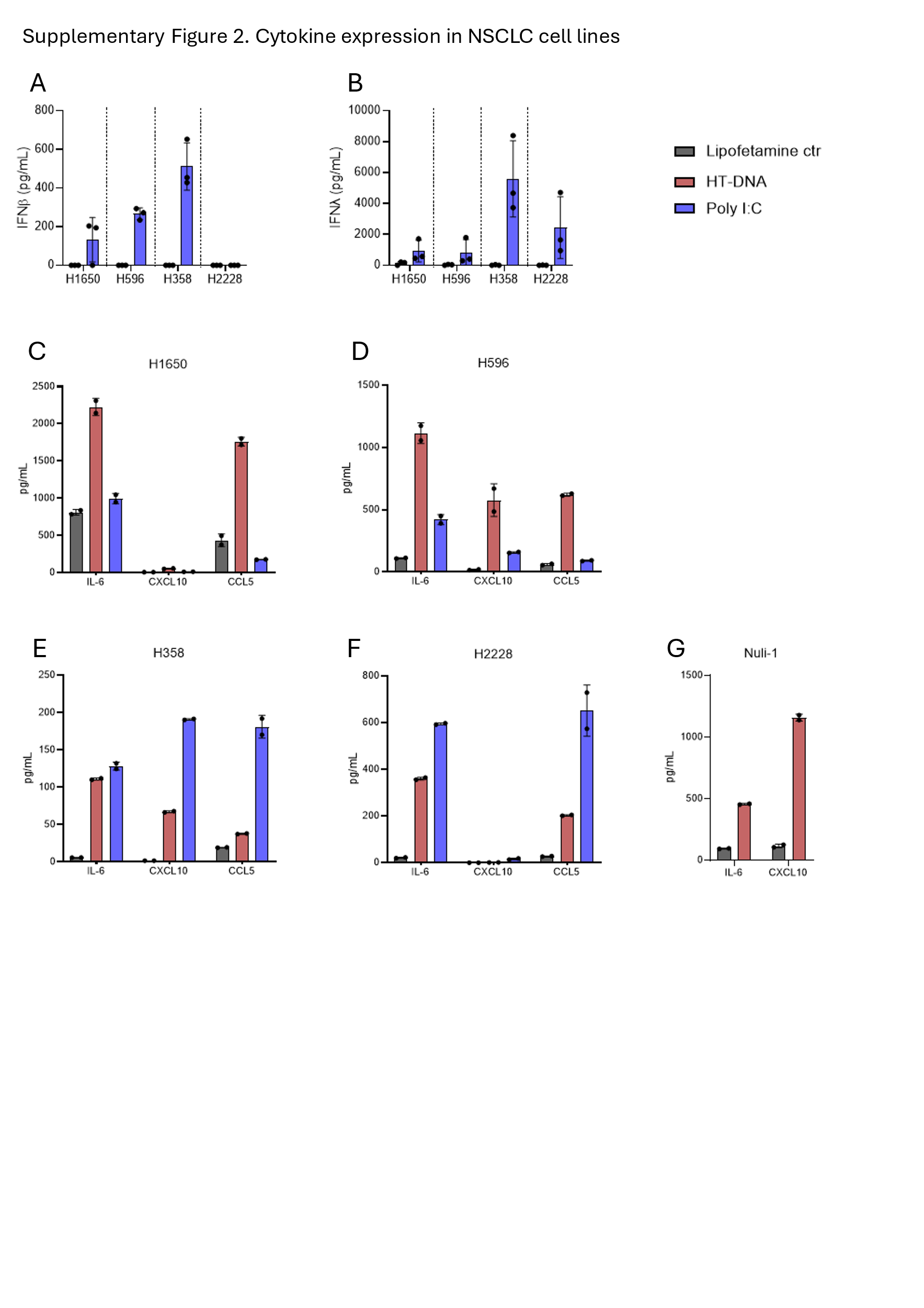


**Supplementary Figure 2. Cytokine expression in NSCLC cell lines.** (**A-B**) IFNβ and IFNλ expression in supernatants from four NSCLC cell lines measured using ELISA. Cells are transfected for 20 hours with Poly I:C (40ng/mL) or a control. Mean +/- standard deviation of three individual experiments is shown. (**C-G**) IL-6, CXCL10, and CCL5 expression in supernatants from four NSCLC cell lines and Nuli-1 cells measured using multiplex or single ELISA. Cells are transfected for 20 hours with HT-DNA (2μg/mL), Poly I:C (40ng/mL), or a control. Mean +/- standard deviation of one experiment performed in duplicates is shown.


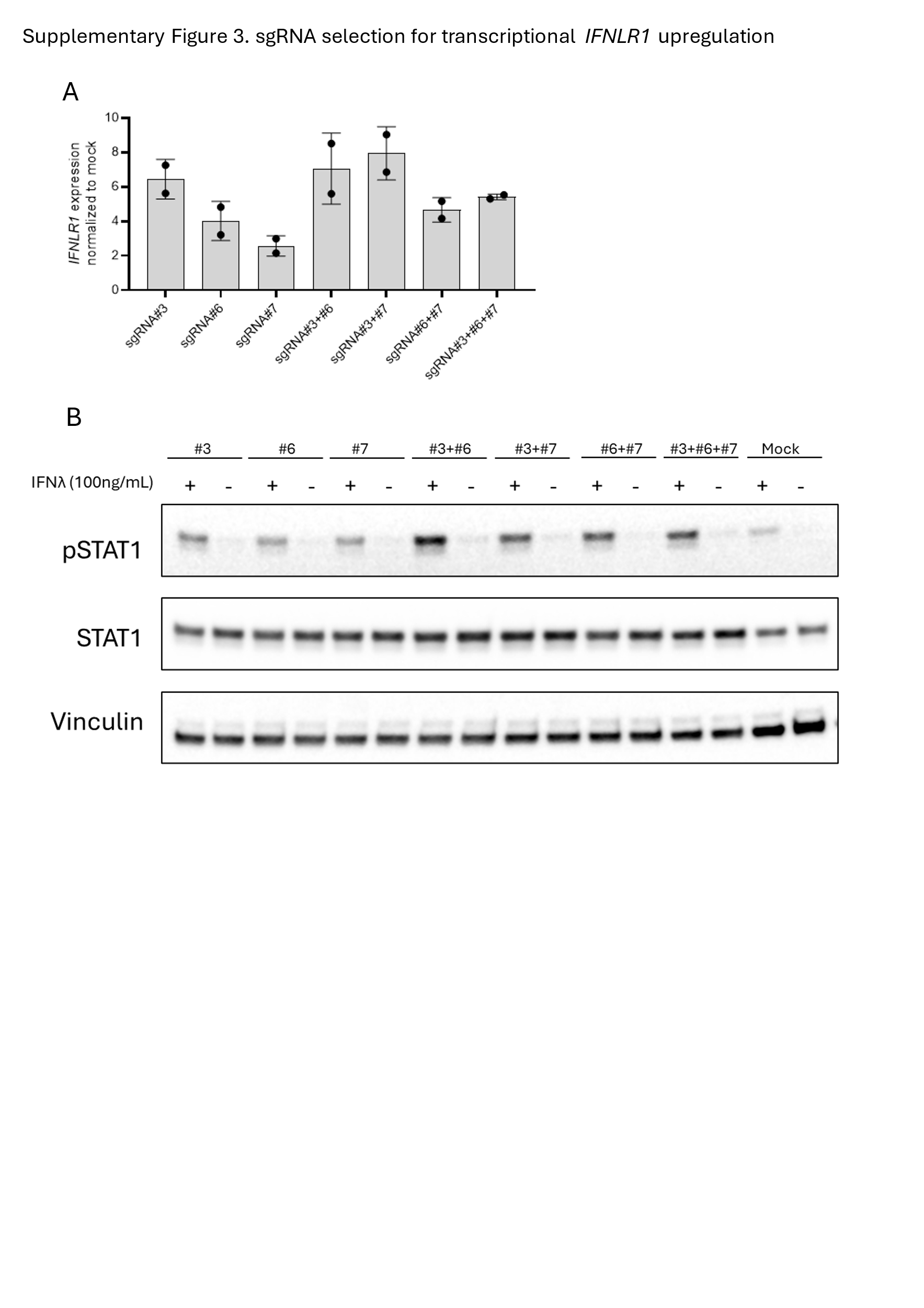


**Supplementary Figure 3. sgRNA selection for transcriptional *IFNLR1* upregulation.** (**A**) *IFNLR1* expression in H1650 cells after CRISPRa with the indicated sgRNAs. Expression levels are normalized to H1650 mock control cells in which transcriptional activation was not performed. Mean +/- standard deviation of duplicates from one experiment is shown. (**B**) western blot of STAT1 and pSTAT1 expression in H1650 cells. Cell lysates were harvested one day after CRISPRa of *IFNLR1* with the indicated sgRNA or combination of sRNAs. Cells were treated 15 min. prior to harvest with IFNλ (100ng/mL) or left untreated. H1650 mock control cells in which transcriptional activation was not performed were included. Vinculin was used as a loading control.


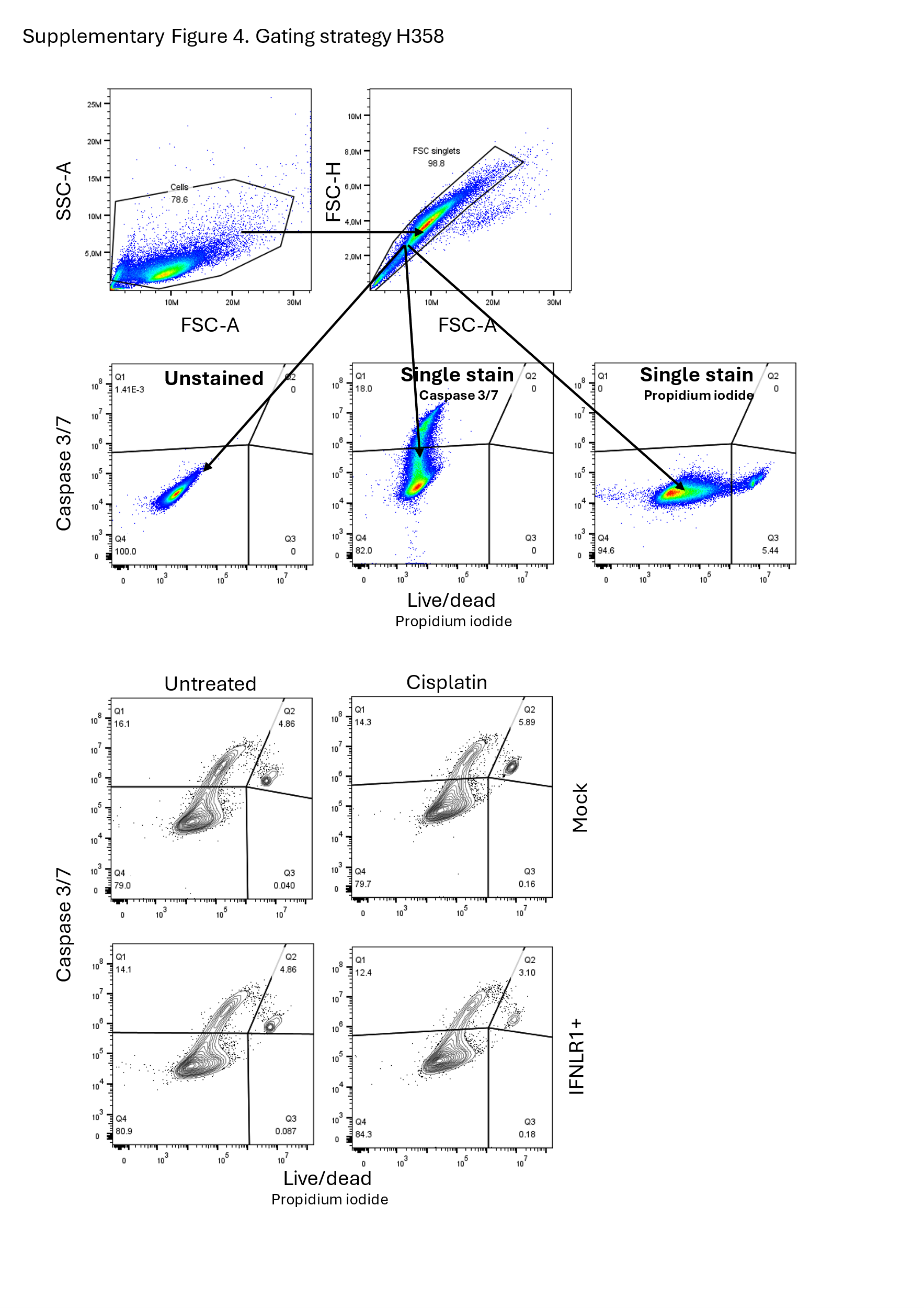


**Supplementary Figure 4. Gating strategy H358.** FSC-A and SSC-A were used to identify H358 cells. Using FSC height versus area, the singlets were gated. Dead/live cells and caspase negative/positive cells were subsequently identified using single stain controls with propidium iodide and caspase 3/7 reagent.


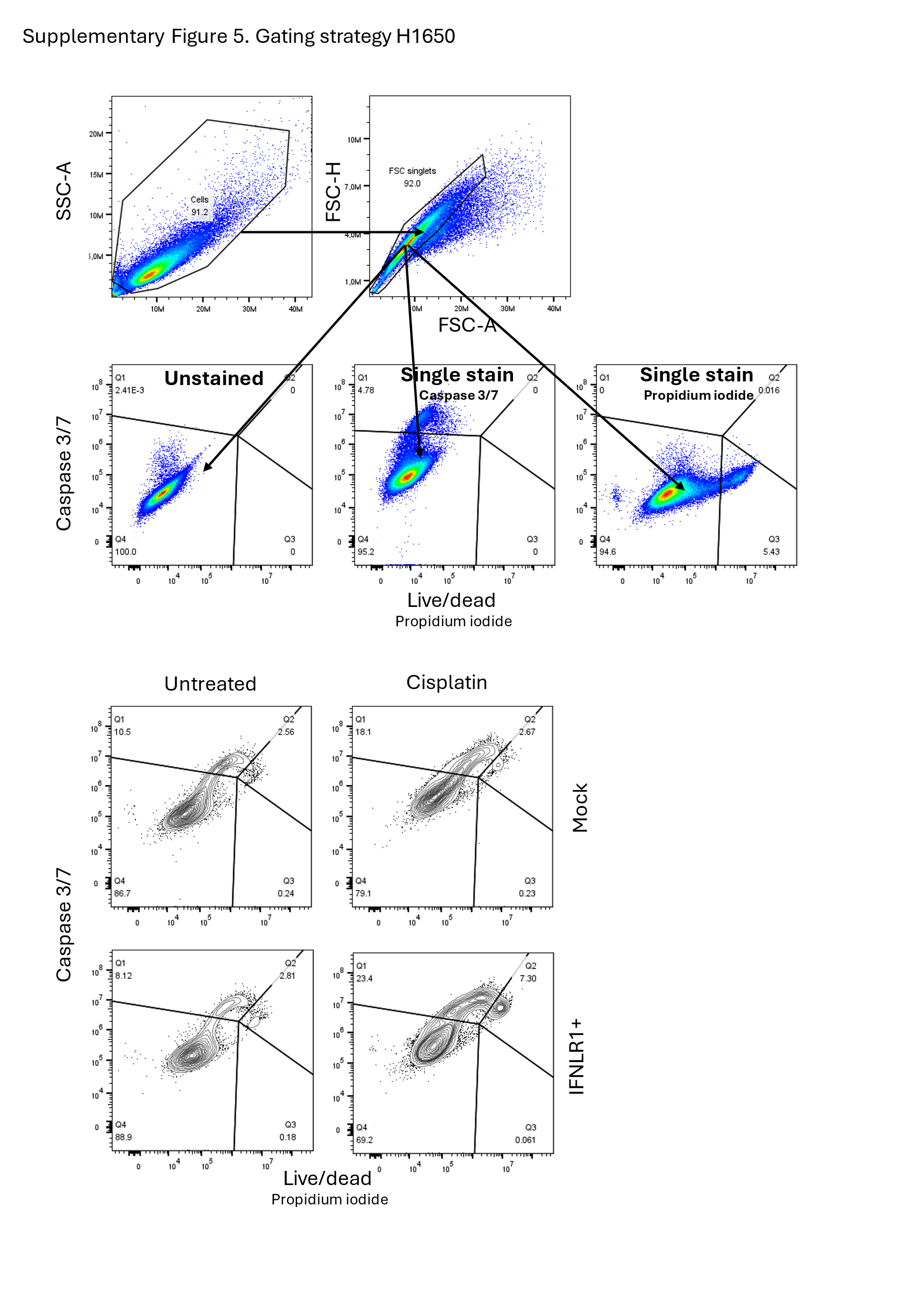


**Supplementary Figure 5. Gating strategy H1650.** FSC-A and SSC-A were used to identify H1650 cells. Using FSC height versus area, the singlets were gated. Dead/live cells and caspase negative/positive cells were subsequently identified using single stain controls with propidium iodide and caspase 3/7 reagent.
