# Supplementary material for: IFNλ1 is a STING-dependent mediator of DNA damage and induces immune activation in lung cancer
Source: Front Immunol. 2025 Feb 12;15:1525083. doi: 10.3389/fimmu.2024.1525083 (PMC11862833; doi:10.3389/fimmu.2024.1525083)
Supplement: Supplementary file 2 [file Table1.docx]

Supplementary Table 1: Gene names and average log2 fold change

| ***Name*** | ***Average log2 fold change*** |
| --- | --- |
| *IFIT2* | 6.27 |
| *IFNL1* | 5.90 |
| *OASL* | 5.47 |
| *IFIT1* | 4.99 |
| *IFIT3* | 4.98 |
| *IFNB1* | 4.95 |
| *RSAD2* | 4.90 |
| *IFNL2* | 4.88 |
| *CXCL10* | 4.84 |
| *IL6* | 4.75 |
| *EGR1* | 4.40 |
| *FOS* | 4.34 |
| *HERC5* | 4.32 |
| *CXCL11* | 4.31 |
| *IFIH1* | 4.25 |
| *RAET1L* | 4.16 |
| *ZC3HAV1* | 4.11 |
| *ATF3* | 4.05 |
| *CH25H* | 3.86 |
| *PMAIP1* | 3.58 |
| *OAS2* | 3.47 |
| *KLF4* | 3.45 |
| *RND1* | 3.29 |
| *GBP4* | 3.27 |
| *ISG20* | 3.21 |
| *DHX58* | 3.14 |
| *FZD4* | 3.13 |
| *KRT17* | 3.12 |
| *BBC3* | 3.11 |
| *RHEBL1* | 3.06 |
| *ISG15* | 3.06 |
| *APOL2* | 3.04 |
| *CMPK2* | 3.00 |
| *MX1* | 2.95 |
| *ZFP36L2* | 2.91 |
| *TRANK1* | 2.85 |
| *NFKBIZ* | 2.85 |
| *NOCT* | 2.83 |
| *PLAUR* | 2.82 |
| *PLEKHA4* | 2.81 |
| *ARC* | 2.81 |
| *PPP1R15A* | 2.77 |
| *OAS1* | 2.77 |
| *CXCL8* | 2.75 |
| *SAMD9* | 2.73 |
| *EGR4* | 2.72 |
| *DDX60* | 2.71 |
| *CXCL2* | 2.70 |
| *MX2* | 2.69 |
| *PTGER4* | 2.69 |
| *GBP5* | 2.68 |
| *NR4A2* | 2.66 |
| *MXD1* | 2.63 |
| *NEURL3* | 2.61 |
| *CEACAM1* | 2.58 |
| *ZFP36* | 2.58 |
| *HERPUD1* | 2.58 |
| *PTGS2* | 2.57 |
| *USP18* | 2.57 |
| *CXCL1* | 2.53 |
| *TNFAIP3* | 2.51 |
| *CHAC1* | 2.49 |
| *HIP1R* | 2.46 |
| *JAK2* | 2.45 |
| *TNFSF10* | 2.41 |
| *IFI44* | 2.41 |
| *OTUD1* | 2.40 |
| *IRF1* | 2.40 |
| *AIM2* | 2.33 |
| *LINC02416* | 2.33 |
| *FOSB* | 2.30 |
| *APOL6* | 2.29 |
| *PPM1K* | 2.26 |
| *APOL1* | 2.24 |
| *JUN* | 2.23 |
| *ZNFX1* | 2.22 |
| *KLF2* | 2.21 |
| *PARP14* | 2.19 |
| *IDO1* | 2.15 |
| *BATF2* | 2.14 |
| *NFKBIA* | 2.13 |
| *DUSP1* | 2.11 |
| *HERC6* | 2.05 |
| *THEMIS2* | 2.04 |
| *EPSTI1* | 2.04 |
| *XBP1* | 2.02 |
| *DDIT3* | 2.00 |

Supplementary Table 2: guide RNA sequences used for CRISPR mediated gene knockout

| ***Target gene*** | ***sgRNA#1*** | ***sgRNA#2*** |
| --- | --- | --- |
| *STING* | CATATTACATCGGATATCTG | ACACTGCAGAGATCTCAGCT |

Supplementary Table 3: Primers for RT-qPCR

| ***Target gene*** | ***Forward primer*** | ***Reverse primer*** |
| --- | --- | --- |
| *IFNB1* | TGCTCTCCTGTTGTGCTTCT | AGCCTCCCATTCAATTGCCA |
| *IFNL1* | GGTGACTTTGGTGCTAGGCT | GGAAGACAGGAGAGCTGCAA |
| *IFNL2* | GGTGACAGCCTCAGAGTGTT | GGGACTTGAACTGGGCTATGT |
| *IFNLR1* | CAACAAGTTCAAGGGACGCG | AGCTGGTACGTGGCATTGG |
| *YWHAZ* | CGTTACTTGGCTGAGGTTGC | ACACAGAGAAGTTAAGGGCCAG |
| *EIF2B2* | CAGAGAGGGCAGGAGGATGA | CCTGCTGATCACTCTCGTCG |

Supplementary Table 4: Simplified and original cell type used in single cell RNA sequencing analysis

| ***Cell type simplified*** | ***Cell type original*** |
| --- | --- |
| Dendritic cell | dendritic cell |
| Dendritic cell | plasmacytoid dendritic cell |
| Dendritic cell | conventional dendritic cell |
| Dendritic cell | CD1c-positive myeloid dendritic cell |
| Epithelial cell | epithelial cell of lung |
| Epithelial cell | multi-ciliated epithelial cell |
| Epithelial cell | type I pneumocyte |
| Epithelial cell | type II pneumocyte |
| Epithelial cell | club cell |
| Macrophage | macrophage |
| Macrophage | alveolar macrophage |
| Malignant epithelial cell | malignant cell |
| Monocyte | classical monocyte |
| Monocyte | non-classical monocyte |
| Neutrophil | neutrophil |

Supplementary Table 5: guide RNA sequences used for CRISPR mediated transcriptional activation of *IFNLR1*

| ***sgRNA#*** | ***sgRNA sequence*** |
| --- | --- |
| 1 | CCGCUCCAGGUAAGGGCGCG |
| 2 | ACGCCGCGGCAGGAAGGCCA |
| 3 | UCAGUGAAUCCUAGGCGGCA |
| 4 | GUGGGUUAUUGAUCAUCAGC |
| 5 | AUCAGUAAAUCCGUGCACGG |
| 6 | GUACGGGCGAGUUUCGAGCG |
| 7 | CCGACUGCGUCACCUGCCCG |
| 8 | GGAGGCGGGAGGCGGGAGGC |
